# Molecular Diagnosis of Putative Stargardt Disease by Capture Next Generation Sequencing

**DOI:** 10.1371/journal.pone.0095528

**Published:** 2014-04-24

**Authors:** Xiao Zhang, Xianglian Ge, Wei Shi, Ping Huang, Qingjie Min, Minghan Li, Xinping Yu, Yaming Wu, Guangyu Zhao, Yi Tong, Zi-Bing Jin, Jia Qu, Feng Gu

**Affiliations:** 1 School of Ophthalmology and Optometry, Eye Hospital, Wenzhou Medical University, State Key Laboratory Cultivation Base and Key Laboratory of Vision Science, Ministry of Health and Zhejiang Provincial Key Laboratory of Ophthalmology and Optometry, Wenzhou, Zhejiang, China; 2 Department of Ophthalmology, Beijing Children's Hospital, Capital Medical University, Beijing, China; 3 Department of Development and Planning, Wenzhou Medical University, Wenzhou, Zhejiang, China; 4 Institute of Genomic Medicine, Wenzhou Medical University, Wenzhou, Zhejiang, China; 5 Department of Ophthalmology, The First Affiliated Hospital of Wenzhou Medical University, Wenzhou, Zhejiang, China; 6 Fuzhou Southeastern Eye Hospital, Fuzhou, China; Purdue University, United States of America

## Abstract

Stargardt Disease (STGD) is the commonest genetic form of juvenile or early adult onset macular degeneration, which is a genetically heterogeneous disease. Molecular diagnosis of STGD remains a challenge in a significant proportion of cases. To address this, seven patients from five putative STGD families were recruited. We performed capture next generation sequencing (CNGS) of the probands and searched for potentially disease-causing genetic variants in previously identified retinal or macular dystrophy genes. Seven disease-causing mutations in *ABCA4* and two in *PROM1* were identified by CNGS, which provides a confident genetic diagnosis in these five families. We also provided a genetic basis to explain the differences among putative STGD due to various mutations in different genes. Meanwhile, we show for the first time that compound heterozygous mutations in *PROM1* gene could cause cone-rod dystrophy. Our findings support the enormous potential of CNGS in putative STGD molecular diagnosis.

## Introduction

Stargardt disease (STGD) is the most frequent cause of macular degeneration in childhood, with a prevalence of approximately 1∶10000 [1]. It is usually diagnosed within the first two decades of life and leads to progressive irreversible loss of central vision, delayed dark adaptation and a poor final visual outcome. STGD is predominantly inherited as an autosomal recessive trait with mutations in *ABCA4, also known as ABCR*, although an autosomal dominant form has been also reported [2]. Rare cases of STGD or “Stargardt-like” disease phenotypes have been reported with mutations in *PROM1*, *PRPH2*, *VMD2* (**also known as** *BEST1*) and *ELOVL4*, which are involved in various physiological pathways that are important for macular function [3]. This complex arena of genes and clinical features complicates the nomenclature in this field [3]; it is unclear how to classify individuals with classic Stargardt phenotype. Classic STGD should be restricted to only those cases caused by *ABCA4* mutations and “Stargardt-like” or juvenile macular dystrophy should be used for other genetic etiologies. For the purposes of this study, we classify our participants with early-onset macular degeneration as “putative STGD” cases.

Stem cell-based therapy shows great promise for the treatment of STGD [4] Accurate molecular diagnosis is therefore essential for the selection of patients for clinical trials, and is also crucial for prenatal STGD diagnosis. However, the genetic diagnosis of individuals with putative STGD is an ongoing challenge because of the relatively large sizes of some of the genes involved. *ABCA4* and *PROM1* are particularly large containing 50 and 26 exons, respectively. *VMD2, ELOVL4 and PRPH2* have 8, 6, and 2 exons, respectively.

Furthermore, although biallelic mutations in *ABCA4* are found in most patients with autosomal recessive STGD, there are studies which have shown that mutations in the *ABCA4* gene are responsible for a wide variety of other retinal dystrophy phenotypes, such as cone-rod dystrophy (CRD), and retinitis pigmentosa (RP) [5], [6]. It has also been proposed that individuals carrying mutations in *ABCA4* may have a higher risk of developing age-related macular degeneration (AMD) [1], [7]. We therefore sought to investigate whether other retinal disease genes besides these reported five genes could lead to putative STGD.

In this study we initially selected known retinal disease genes as a gene capture panel and applied a capture next generation sequencing (CNGS) approach to identify genetic defects in seven putative STGD patients from five independent families. This approach was used to test whether additional retinal disease genes could lead to putative STGD.

## Materials and Methods

### Patient Recruitment

**Figure 1:**
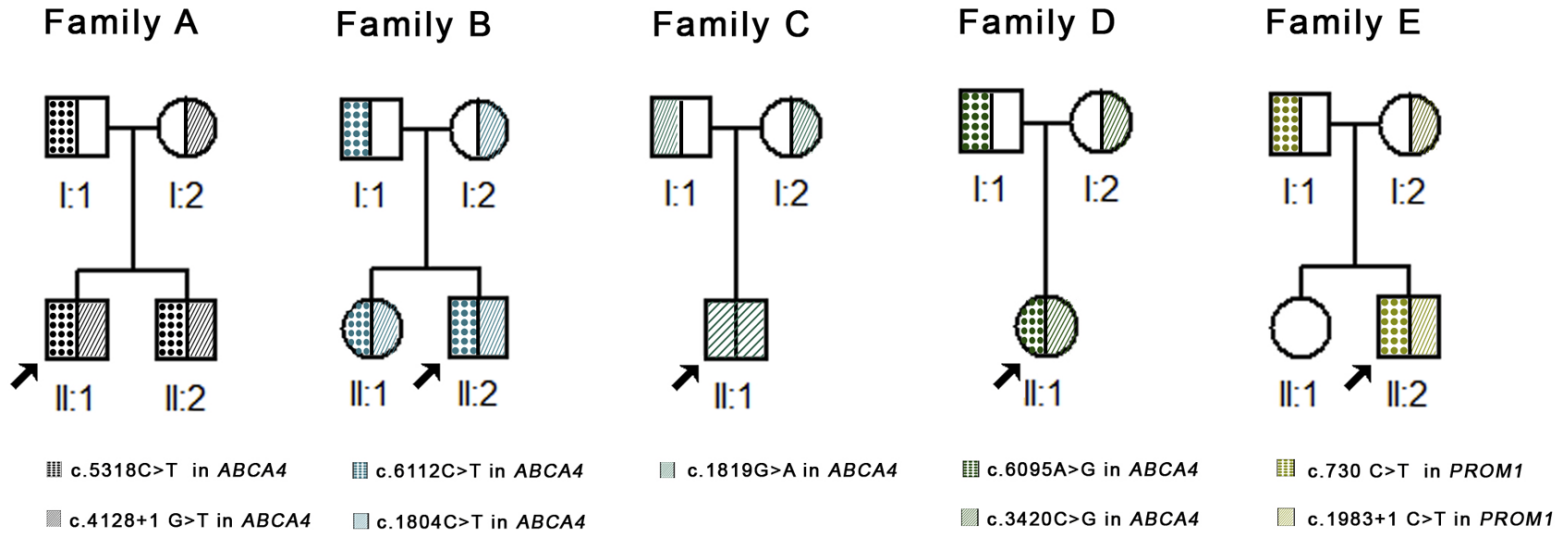
Putative Stargardt disease families and genotype. Pedigrees of the putative STGD families in this study. Filled, half-filled and unfilled symbols denotes affected, carrier and unaffected status, respectively. Arrow indicates proband. The mutations originated from paternal or maternal allele were defined.

This study conformed to the tenets of the Declaration of Helsinki and was approved by the Ethics Committee of Eye Hospital, Wenzhou Medical University. Written informed consent was obtained from all participating individuals or their guardians. Patients from families (A, B, C, D and E, Figure 1) were recruited. Ophthalmic examination was performed for each patient. Electroretinography (ERG) and optical coherence tomography (OCT) were performed as routine retinal ophthalmic examination. A five ml venous blood sample was drawn into an ethylenediamine tetraacetic acid (EDTA) sample tube from every subject. Genomic DNA was extracted from peripheral blood leukocytes using the standard phenol/chloroform extraction protocols.

### Targeted Exome Illumina Library Preparation

Genomic DNA was purified and quantified with Nanodrop 2000 (Thermo Fisher Scientific, DE). The generation of a targeted exome Illumina Library was performed according to the manufacturer's protocol (MyGenostics, Beijing, China). A final library size of 350–450 bp including adapter sequences was selected.

### Disease Genes Enrichment, Sequencing Data Generation and Bioinformatics Analysis

A total of 144 disease genes associated with retinal diseases including the five known STGD genes (*ABCA4*, ***PROM1*** **,** ***PRPH2*** **,** ***VMD2*** **and** ***ELOVL4***) were selected by a gene capture strategy, using the GenCap custom enrichment kit (MyGenostics), as previously described [8]. The enriched libraries were sequenced on an Illumina Solexa HiSeq 2000 sequencer for paired-end reads of 100 bp, and analyzed as described previously [8]. Briefly, using the Solexa QA the cutadapt (http://code.google.com/p/cutadapt/), SOAP aligner, BWA and GATK programs to retrieve and align to identify SNPs and insertions or deletions (InDels). SNPs and InDels were annotated using the exome-assistant program (http://122.228.158.106/exomeassistant). Nonsynonymous variants were evaluated by three algorithms, SIFT (http://sift.jcvi.org/), PolyPhen (http://genetics.bwh.harvard.edu/pph2/) and PANTHER (http://www.pantherdb.org/tools/csnpScoreForm.jsp) as described previously, to determine pathogenicity [13]. Multiple sequence alignments were performed using ESPript3.0 (http://espript.ibcp.fr/ESPript/cgi-bin/ESPript.cgi). Sequencing data were deposited in NIH Short Read Archive (SRP036846).

### Additional Sequencing

Targeted amplification of *ABCA4* and *PROM1* sequences was performed using PCR (primer sequences and amplification conditions in Table S1 in File S1). PCR products were sequenced on an ABI PRISM 3730 DNA Sequencer. For mutations, nucleotide numbering reflects cDNA numbering with +1 corresponding to the A of the ATG translation initiation codon in the reference sequence, according to journal guidelines (www.hgvs.org/mutnomen). The initiation codon is codon 1.

## Results

### Clinical Data

**Figure 2:**
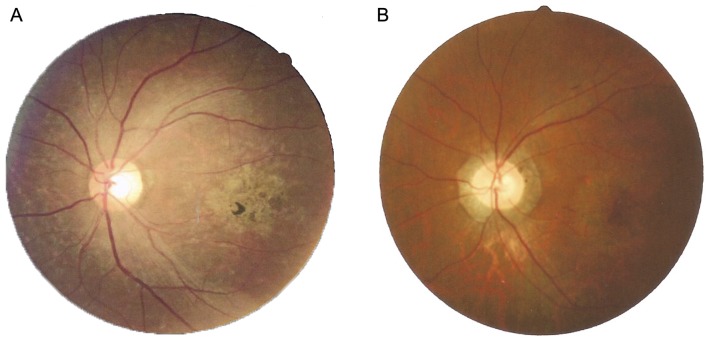
Color Fundus photographs of the patients. Color fundus photograph of patient II:1 from family B and patients II:1 from family E. Both of them showed macular atrophy.

A total of seven patients (two females and five males, Figure 1) from five independent families were recruited for this study. Clinical summaries, including visual acuity, age of recruitment, gender, and relevant ophthalmological findings are described in Table 1, Figure 2 and Figure S1 in File S1. No night-blindness was observed in any of the enrolled patients. We noticed the onset of disease in patients from family E was later than that of other patients, which led us to suspect that it has a distinct genetic etiology.

**Table 1:** Clinical summaries of individuals in this study.

Family	Individual ID	Gender	Age (Y)	Age (Y) at onset	BCVA at presentation	Color-vision problems
A	III:1	M	21	10	20/200	N/A*
	III:2	M	13	11	20/200	+*
B	II:1	M	34	10	20/250	+
	II:2	F	31	13	20/400	+
C	III:1	M	23	10	20/200	+
D	II:1	F	13	6	20/200	N/A
E	II:1	M	25	17	20/200	+

M,Male; F,Female; BCVA, Best corrected visual acuity; N/A*, No Answer; +*, Affected.

### Mutation Analysis

**Figure 3:**
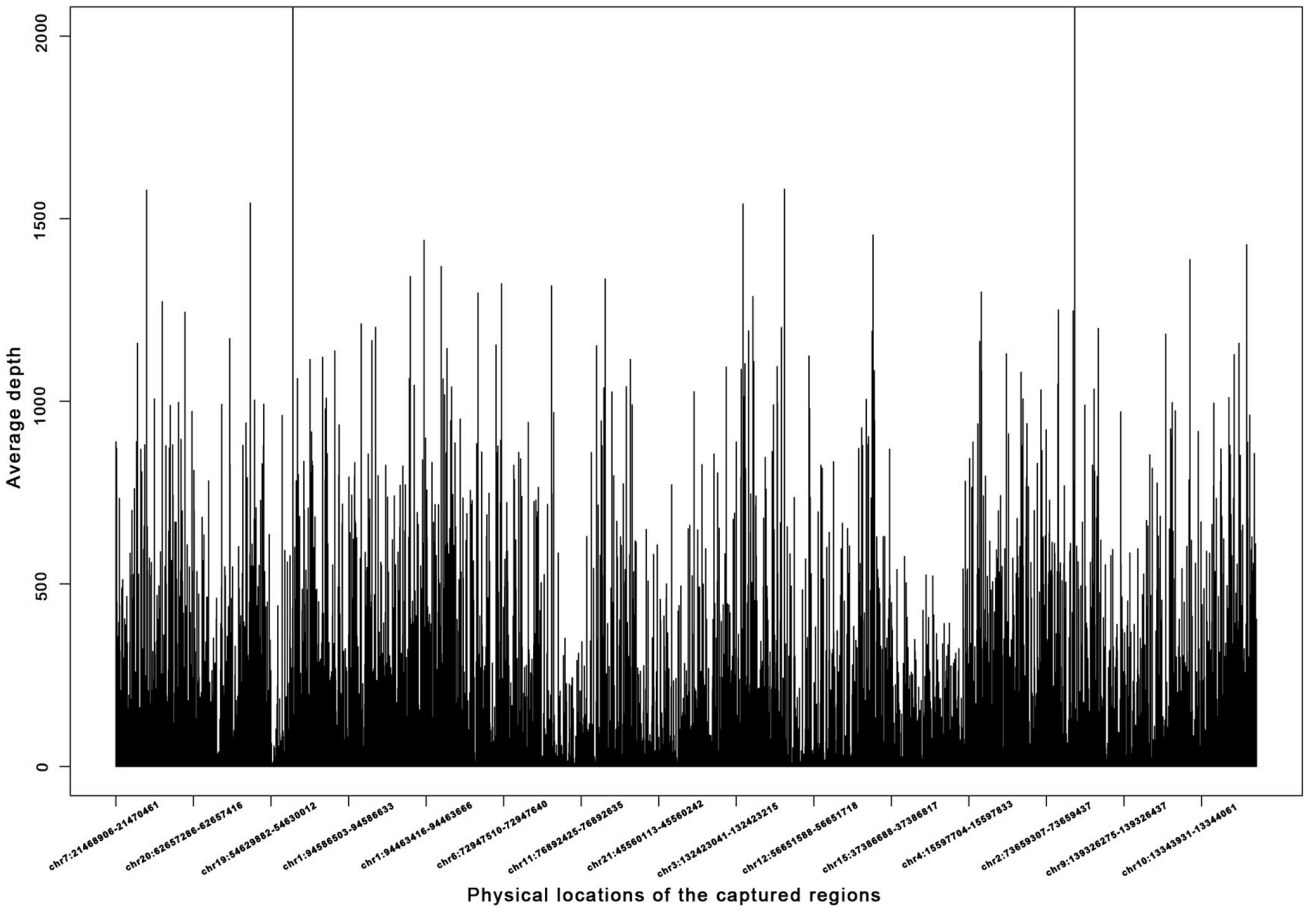
Mean coverage of Capture Next Generation Sequencing. A mean coverage of 200× over the targeted region was achieved; part of the sequencing region was showed.

We first calculated the CNGS results for quality. On average, a mean coverage of 200× over the targeted region was achieved (Figure 3). Manual checking of sequencing depth of known STGD genes (*ABCA4*, *PROM I*, *PRPH2*, *VMD2* and *ELOVL4*) (Table S2 in File S1) showed that a mean coverage of 216× was obtained. We observed that missing coverage of one exon each in *PROM1* (Exon24) and *VMD2* (Exon4) genes. We used Sanger sequencing for these two missing exons (primer sequences shown in Table S1 in File S1).

Given that autosomal recessive STGD is largely caused by homozygous or compound heterozygous mutations, we initially scanned the five reported STGD genes for mutations in our dataset. Sanger sequencing was used for validation of any pathogenic mutations identified, and the segregation of mutations was tested in the familial cases. For any missense variants identified, computational prediction by three algorithms was used to confirm the number of candidate mutations.

**Figure 4:**
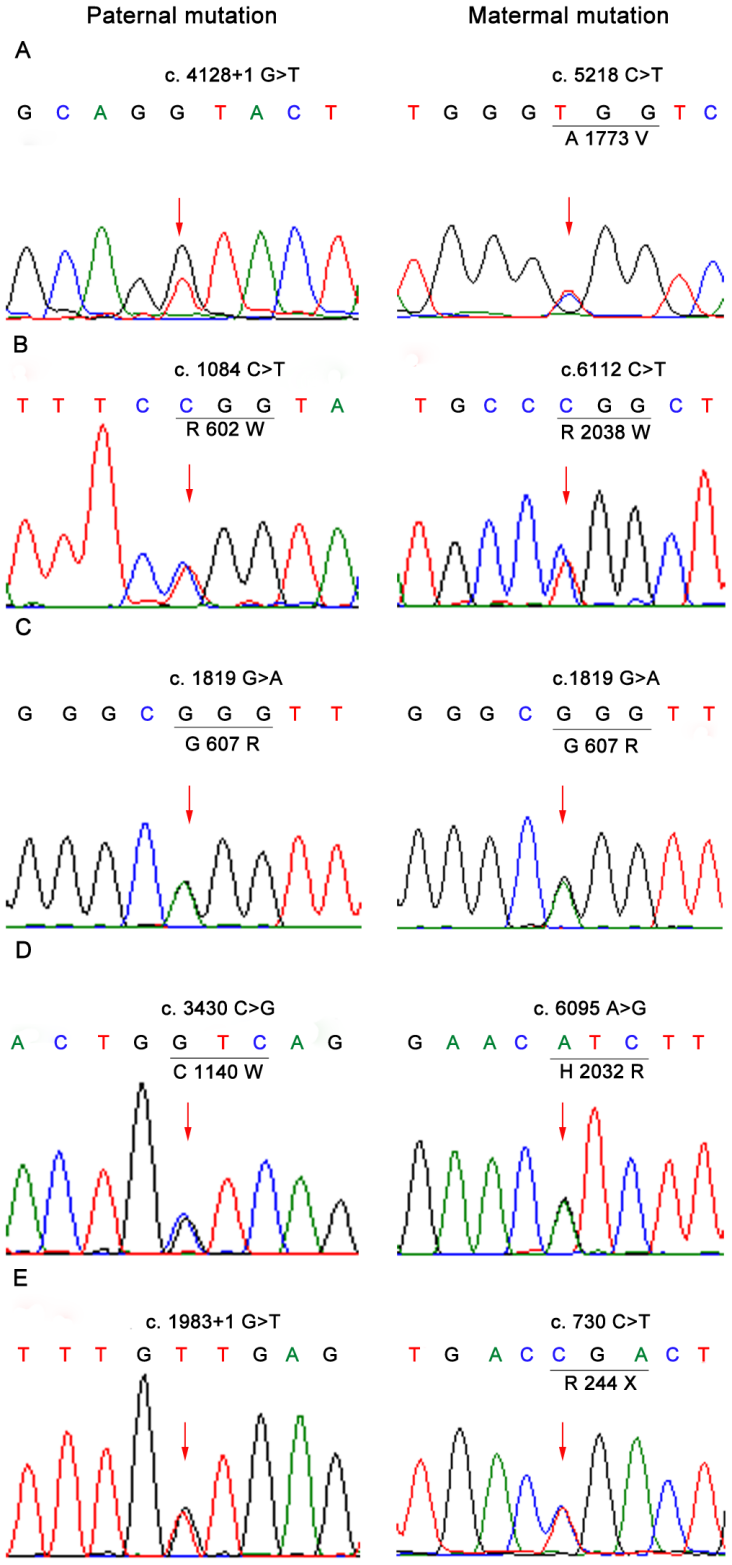
DNA sequence chromatograms. DNA sequence chromatograms of the affected members with putative Stargardt disease. The heterozygous peaks of the mutations were pointed out by red arrow. The origin of each mutation was being traced from paternal or maternal allele.

**Figure 5:**
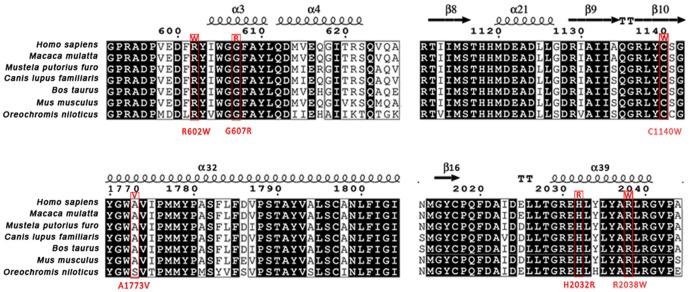
Multiple-sequence alignment of ABCA4 from different species. Multiple-sequence alignment in ABCA4 from different species revealed that these mutations were located within a highly conserved region. The mutations were highlighted by red box. Majority of the identified mutations (4 of 6) were located within the alpha-helix regions. α-helices are displayed as squiggles; β-strands as arrows; strict β-turns as TT letters.

The mutations identified in these cases are summarized in Table 2. Briefly, seven mutations (one homozygous) in *ABCA4* and two mutations in *PROM1* were successfully identified in the STGD families via CNGS, Sanger sequencing, and co-segregation analysis (Figure 4 and Figure 1, Figure S2 in File S1 and Table S1 in File S1). Furthermore, multiple sequence alignments were performed and we found that missense mutations in *ABCA4* were located within a phylogenetically conserved region (Figure 5).

**Table 2:** Identified mutations summary.

								Allele frequency	
Family	Gene	Identified Mutations (Exon)	Reported or novel	SIFT	PolyPhen	PANTHER	1000G	ESP6500	In-house
A	*ABCA4*	c.5318C>T;p.A1773V (Exon 38)	Reported	D$	PD$	T$	0	0	0
		c.4128+1 G>T (Exon 27)	Novel	N/A	N/A	N/A	0	0	0
B	*ABCA4*	c.6112C>T; p.R2038W (Exon 44)	Reported	D$	PD$	De$	0	0.00008	0
		c.1804C>T; p. R602W (Exon 13)	Reported	D$	Benign	De$	0	0	0
C	*ABCA4*	c.1819G>A;p.G607R(Exon 13, Homo*)	Reported	D$	PD$	De$	0	0.000077	0
D	*ABCA4*	c.6095A>G; p.H2032R (Exon 44)	Novel	D$	PD$	De$	0	0	0
		c.3420C>G;p.C1140W (Exon 23)	Novel	D$	PD$	De$	0	0	0
E	*PROM1*	c.730C>T; p.R244X (Exon 6)	Novel	N/A	N/A	N/A	0	0	0
		c.1983+1 C>T (Exon18)	Novel	N/A	N/A	N/A	0	0	0

$ D: Damaging; PD, Possibly damaging; T, Tolerated; DE,Deleterious; N/A, No Answer;

*Homo, Homozygous mutation; , SIFT (http://sift.jcvi.org/); PolyPhen (http://genetics.bwh.harvard.edu/pph2/).

PANTHER (http://www.pantherdb.org/tools/csnpScoreForm.jsp); 1000G (http://www.1000genomes.org/);

ESP6500 (http://evs.gs.washington.edu/EVS/); In-house, in-house exome database.

With the exception of family C all families had compound heterozygous mutations. Additionally, because we can define whether the mutation originated from paternal or maternal allele, we could trace the origin of each mutation (Figure 1, Figure 4). No *de novo* mutations were identified in these families.

We searched for the mutations identified in multiple databases, including the 1000 Genome (1000G, http://www.1000genomes.org/), ESP6500 (http://evs.gs.washington.edu/EVS/) and 702 sample in-house exome database as normal controls. The candidate mutations were not present or present at extremely low frequency in these databases (Table 2). The molecular genetics of these families clearly shows that patients with *PROM1* mutations have later onset disease, compared with patients with *ABCA4* (17-years-old vs 6 to13-years-old).

We found that in our cohort of putative STGD patients that no mutations in additional retinal disease genes besides these reported five genes were identified. We did observe additional heterozygous DNA variants (Table S3 **in File S1**).

### Post-Identified-Mutations for the Clinical Diagnosis

**Figure 6:**
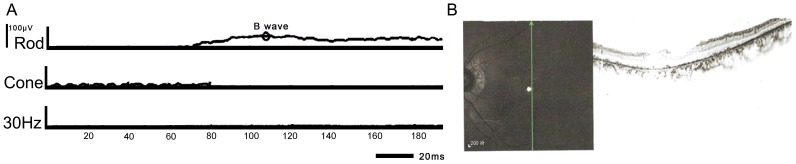
Electroretinogram and OCT testing of the patient from family E. A. The electroretinogram (ERG) showed cone responses are more severely affected than rod responses (Only B wave has a small peak at 108 ms/56.6uv). This is the key clinical characteristics for diagnosis of CRD. B. OCT (Optical Coherence Tomography) testing of the patient from family E showed that photoreceptor segments and the retinal pigment epithelium were seriously affected.

After the mutations were identified in families, we realized that re-evaluation of patients was necessary especially for family E because there are no reports that compound heterozygous *PROM1* mutations have been identified for putative STGD. We retrieved the clinical data from these families and confirmed that the diagnosis of patients from family A–D is STGD. In Family E, we found retinal vessels of the patients are moderately attenuated (Figure 2), and the macular atrophy was very obvious. The electroretinogram (ERG) showed that both the cone and rod responses were affected; meanwhile, the cone responses were more severely affected than rod responses (Figure 6 A). OCT (Optical Coherence Tomography) testing showed the photoreceptor segments and the retinal pigment epithelium atrophy seriously (Figure 6B). Visual field testing showed central scotomas, while the periphery was spared (Figure S3 **in File S1**). The Arden ratios of the electro-oculogram (EOG) were 1.2 and 1.5 (Figure S4 **in File S1**), respectively, compared with that of normal (1.8). Taken together, based on the clinical manifestations, the final diagnosis of patients from family E was cone-rod dystrophy (CRD). This highlights that accurate clinical diagnoses should based on all the available clinical data because there are substantially overlapping phenotypes between STGD and CRD (Table S4 **in File S1**). Also, it demonstrates that the CNGS method is indeed the best method to determine the genetic cause of a heterogeneous disease since it is unbiased. It is also the case that CNGS method may be helpful for the accurate clinical diagnoses of heterogeneous diseases even if the researcher does not have access to clinically well characterized patients with different forms of retinal disorders.

## Discussion

STGD is the most common childhood recessively inherited macular dystrophy. The first identified disease gene linked with STGD was *ABCA4* in 1997 by *Allikmets et al.* [9], and since then, many additional mutations have been identified [1]–[3], [7], [10]. Here we recruited five families and found that four of them have *ABCA4* mutations, which indicates that it is very informative to screen for *ABCA4* mutations in STGD families.

So far, the use of a variety of mutation detection techniques for STGD such as SSCP (single-strand conformation polymorphism)/heteroduplex analysis, high resolution melting, microarray, direct Sanger sequencing and PCR-Next-Generation Sequencing (PNGS), and whole exome sequencing (WES) approaches have been reported [2], [3], [6], [10]–[15]. With the exception of PNGS and WES these methods are labor **intensive** or low throughput approaches. Although the PNGS method has the advantage of high throughput, it may be a challenge to amplify of all the reported gene fragments in one tube. The bioinformatics analysis of the results from WGS is still challenging for most laboratories and the cost may be prohibitive. In contrast, CNGS allows for the comprehensive molecular diagnosis of these heterogeneous genetic diseases and has the advantages of speed (Exons of 144 disease genes sequenced at one time) and is cost-effective (less than 1/40 cost of Sanger sequencing); here we demonstrated the usefulness of this approach.

We observed missing coverage of some exons from CNGS based molecular diagnosis of STGD, which indicates the method still has flaws for applications in clinical genetic diagnosis. It has been reported that with deep sequencing, coverage of some regions will be missing [16], [17]. The main reason for this may be the PCR step, which has a bias for amplification of GC-rich or repeat fragments under normal PCR conditions. We analysed the missing coverage of exons in two genes (*PROM1* and *VMD2*), then found there is a repeat A sequence in *PROM1* and more than 60% GC content in the corresponding exon of *VMD2*. To fill in the missing data, we designed specific primers to amplify these fragments. The results from the present study also suggest that before searching for the disease-associated mutation, it is necessary to check the coverage of the targeted sequence, even though no mutations were found in the missing coverage region.

Definition of a “disease-associated” mutation is a difficult task, particularly if no simple functional assays to determine the phenotypic effects of specific variations are readily available [1]. In general we use the following criteria: if the mutation allele frequency is over 5% in the general population as identified by bioinformatics analysis of multiple databases, we would treat it as a non-pathogenic mutation since STGD prevalence of approximately 1∶10000. We also checked it whether the mutation was reported in the literature or is novel. In this study, we identified these nine mutations, including five novels and four previously reported, which expands the mutation spectrum of *ABCA4* and *PROM1*.

Analysis of disease **allele frequency** in specific populations is important for clinical genetic diagnosis. There are reports that “population-specific” *ABCA4* alleles, such as p.G863A/delG863, are founder mutations in Northern European patients. We searched all these nine mutation in the literature and found five novels and four previously reported. Among these four reported mutations, p.A1773V in *ABCA4* was reported as one of the founder mutations (up to17%) in Latin American population [18]; p.R2038W mutation in USA, Estonia and South African population; p.R602W mutation in USA, South African population [2], [3], [19]; G607R in the German population[20]. Taken together, this study confirmed that these four mutations are pathogenic mutations and among these four reported mutations, p.A1773V, p.R2038W and p.R602W may have higher allele frequencies since they were frequently reported in different populations. We observed two mutations (p.R2038W and p.G607R) [1], [2], which have extremely low allele frequency (0.000080 and 0.000077, respectively), in databases (Table 2), while no allele frequency data (just <1/1404) is available in the Chinese population due to the relatively small in-house sample size. As to real allele frequency of the mutations identified in this study, further studies are needed. This is consistent with the prevalence of STGD (approximately 1∶10000) since the allele frequency of all the mutations identified in this study is not detected in our 702 sample in-house exome database. We speculate that the p.G607R mutation may have a higher allelic frequency, because the patient from family C has homozygous mutation of p.G607R and the parents come from different regions. One previous study of STGD in the Chinese population, screened part of *ABCA4* coding sequence (15 exons) and identified two relatively common mutations: T1428M and R2040X [21]. To further clarify *ABCA4* mutation spectrum in the Chinese population, further studies of large sample size are still needed.

So far, more than 200 disease-associated *ABCA4* variants have been identified (http://www.uniprot.org/uniprot/P78363). We manfully mapped these mutations to the ABCA4 protein and found the majority of the mutations are located at extracelluar and intracellular loops (Figure S5 in File S1). There are four mutation-rich regions at the protein level, which suggests that they are in a key functional region of ABCA4 (Figure S6 in File S1). For genetic diagnosis, it is meaningful to scan these mutation-rich exons. Therefore, we manfully mapped all the mutations and found five exons (3, 13, 22, 29 and 47) have more mutations per length than other exons (Figure S7 in File S1). This indicates that these exons may be prioritized for the detection of mutations in *ABCA4*.

From our five families study, it is clear that patients with *PROM1* mutations have a later age of onset, compared the patients with *ABCA4* mutations. This may be a clinically relevant observation. We reviewed the literature and found mutations in *PROM1* can lead to several diseases (Table 3), including Stargardt disease (STGD4) [22], [23], retinitis pigmentosa (RP41) [24]–[28], autosomal dominant cone-rod dystrophy (CORD12, CRD) [16], macular dystrophy (MCDR2) [29] and autosomal recessive cone-rod dystrophy (CRD) [21]. The major difference between these four diseases (STGD, CRD, MCDR2 and RP41) is the results of full field ERG and fundus appearance, while night-blindness, the ages of onset may be helpful for the clinical diagnosis. CRD is a panretinal photoreceptor degenerative disorder with predominant loss of cone function that affects the macula early in its course, while STGD is a progressive bilateral atrophy of the retinal pigment epithelium in the macula, with accumulation of a lipofuscin-like substance in the retinal pigment epithelium, and a reduced foveal cone ERG. Full field ERG is the key test, particularly when patients are asymptomatic and show a normal fundus at early stages because full field ERG examination can distinguish the effect of degree of cone function and rod function. In other words, cone function in CRD would be affected earlier or more severely than in STGD as measured by the ERG test.

**Table 3:** *PROM1* gene mutations and diseases.

Mutations	Homo/heterozygous	R/D	Disease	Age(Y) at onset	Vision	Clinical findings	Ref.
c.1117C>T ;	Heterozygous	D	STGD4	N/A	20/200	Macular dystrophy	22
p.R373C							
c.1117C>T ;	Heterozygous	D	MCDR2	N/A	20/80	RPE atrophy	22
p.R373C							
c.1117C>T ;	Heterozygous	D	CRD	N/A	N/A	Photoreceptor degeneration	22
p.R373C							
c.1841delG;	Homozygous	R	RP41	Early childhood	N/A	Night blindness	24
p.G614EfsX12*							
c.869delG	Homozygous	R	RP41	N/A	20/100	RPE alteration in the macula area	25
c.1142–1G>A	Homozygous	R	RP41	9	20/125	Discrete central atrophy in the macula	26
c.1726C>T;	Homozygous	R	RP41	Early childhood	20/200	Macular degeneration	27
p.G576X							
c.1349insT;	Homozygous	R	CRD	Early childhood	20/200	Pigment deposits, mild photophobia	28
p.Y452fsX12							
c.730C>T;	Heterozygous	R	CRD	17	20/200	Macular atrophy; RPE atrophy	In this study
p.R244X							
c.1983+1 C>T	Heterozygous	R	CRD	17	20/200	Macular atrophy; RPE atrophy	In this study

R/D: Recessive and Dominant; STGD, Stargardt Disease; MCDR,Macular Dystrophy; CRD, Cone-Rod Dystrophy;

RP, Retinitis Pigmentosa; N/A, No Answer;

*originally as c.1878del G.

Combined the comprehensive clinical examination and genetic diagnosis, this is the first report, to our knowledge, to show that compound heterozygous mutations in *PROM1* could lead to CRD. This study also demonstrates that genetic testing can help to improve the diagnostic accuracy of heterogeneous disease.

In the present study, we demonstrated that no additional retinal disease genes could cause STGD. This may be due to the limited sample size of our study; it also suggests that if additional causative genes for STGD exist, that they may be present in a relative small fraction of cases. Since there is no more available data from additional similar reports, the real fraction still needs to be investigated. However, we cannot exclude novel genes, beyond the scope of our existing knowledge of retinal diseases. It also indicates that, it is very meaningful to scan the mutation in *ABCA4* gene before screening the all retinal disease genes, since to date mainly *ABCA4* has been the gene underlying this disorder.

In summary, we have demonstrated the utility of CNGS approach to molecular diagnosis of putative STGD in five independent families, with successful identification of disease-causing mutations, including five novel mutations. The study also provides a genetic basis of the differences among putative STGD patients due to different mutations in different genes, which is a very significant advance in clinical genetic diagnosis of putative STGD. We showed that compound heterozygous mutations in *PROM1* could cause cone-rod dystrophy for the first time. Our findings support the enormous potential of CNGS in putative STGD molecular diagnosis. With the progress of next generation sequencing technology, higher sequencing quality will be provided and its cost will dramatically decrease, which are the key bottom-necks for the application of CNGS to clinical genetic testing. Here we only showed CNGS results from five families, and more studies should be performed before its application as a routine clinical genetic testing method.

## Supporting Information

File S1 Contains the following files: **Figure S1.** Color fundus photographs of the patients. Color fundus photograph of probands from family A (A), family C (B) and family D (C). All of images showed bull's-eye maculopathy, which is one of the standards for Stargardt disease diagnosis. **Figure S2.** DNA sequence chromatograms of the controls. DNA sequence chromatograms of the controls. The peaks pointed out by red arrow were the mutation sites identified in this study (here is the wild-type). **Figure S3.** Visual field testing of the patient from family E. Visual field testing showed central scotomas, while the periphery was spared. **Figure S4.** Electro-oculogram of the patient from family E. The decreased Arden ratios of the electro-oculogram (EOG) were 1.2 and 1.5, compared with that of normal (1.8). **Figure S5.** *ABCA4* mutations at protein level. The mutations were mapping to each domains of the ABCA4 protein. Each line in red represents one mutation. Blue lines represent mutation identified in this study. **Figure S6.** *ABCA4* mutations and their relative frequencies at protein level. The mutations were mapped to each domains of the ABCA4 protein. There are four mutation-rich loops (intercellular loop 4, intercellular loop 7 and extracelluar loop 1 and extracelluar loop 5). Here it suggests that they are in a key functional region of ABCA4. **Figure S7.** The mutations in each exon and their relative mutation rate in *ABCA4*-related diseases. We mapped all the ABCA4 mutations to its 50 exons and found five exons (3, 13, 22, 29 and 47) have more mutations per length than that of other exons. **Table S1.** PCR information for the amplication of *ABCA4*,*PROM1* and *VMD2* genes. **Table S2.** Capture Next Generation Sequencing of *ABCA4*, *PROM1*, *PRPH2*, *VMD2* and *ELOVL4* genes. **Table S3.** Additional DNA variants identified in CNGS. **Table S4.** Brief Comparison of Stargardt Disease and Cone-Rod Dystrophy.
(RAR)

## References

[1] Rivera A, White K, Stohr H, Steiner K, Hemmrich N, et al (2000) A comprehensive survey of sequence variation in the ABCA4 (ABCR) gene in Stargardt disease and age-related macular degeneration. Am J Hum Genet 67: 800–813. 10958763 10.1086/303090 PMC1287885

[2] September AV, Vorster AA, Ramesar RS, Greenberg LJ (2004) Mutation spectrum and founder chromosomes for the ABCA4 gene in South African patients with Stargardt disease. Invest Ophthalmol Vis Sci 45: 1705–1711. 15161829 10.1167/iovs.03-1167

[3] Strom SP, Gao YQ, Martinez A, Ortube C, Chen Z, et al (2012) Molecular diagnosis of putative Stargardt Disease probands by exome sequencing. BMC medical genetics 13: 67–75. 22863181 10.1186/1471-2350-13-67 PMC3459799

[4] Schwartz SD, Hubschman JP, Heilwell G, Franco-Cardenas V, Pan CK, et al (2012) Embryonic stem cell trials for macular degeneration: a preliminary report. Lancet 379: 713–720. 22281388 10.1016/S0140-6736(12)60028-2

[5] Maugeri A, Klevering BJ, Rohrschneider K, Blankenagel A, Brunner HG, et al (2000) Mutations in the ABCA4 (ABCR) gene are the major cause of autosomal recessive cone-rod dystrophy. Am J Hum Genet 67: 960–966. 10958761 10.1086/303079 PMC1287897

[6] Klevering BJ, Yzer S, Rohrschneider K, Zonneveld M, Allikmets R, et al (2004) Microarray-based mutation analysis of the ABCA4 (ABCR) gene in autosomal recessive cone-rod dystrophy and retinitis pigmentosa. Eur J Hum Genet 12: 1024–1032. 15494742 10.1038/sj.ejhg.5201258

[7] Fujinami K, Zernant J, Chana RK, Wright GA, Tsunoda K, et al (2013) ABCA4 Gene Screening by Next-Generation Sequencing in a British Cohort. Invest Ophthalmol Vis Sci 54: 6662–6674. 23982839 10.1167/iovs.13-12570 PMC3796939

[8] Huang XF, Xiang P, Chen J, Xing DJ, Huang N, et al (2013) Targeted exome sequencing identified novel USH2A mutations in Usher syndrome families. PloS one 8: e63832. 23737954 10.1371/journal.pone.0063832 PMC3667821

[9] Allikmets R (1997) A photoreceptor cell-specific ATP-binding transporter gene (ABCR) is mutated in recessive Stargardt macular dystrophy. Nat Genet 17: 122. 10.1038/ng0997-122a 9288113

[10] Zernant J, Schubert C, Im KM, Burke T, Brown CM, et al (2011) Analysis of the ABCA4 gene by next-generation sequencing. Invest Ophthalmol Vis Sci 52: 8479–8487. 21911583 10.1167/iovs.11-8182 PMC3208189

[11] Michaelides M, Chen LL, Brantley MA Jr, Andorf JL, Isaak EM, et al (2007) ABCA4 mutations and discordant ABCA4 alleles in patients and siblings with bull's-eye maculopathy. Br J Ophthalmol 91: 1650–1655. 18024811 10.1136/bjo.2007.118356 PMC2095527

[12] Duno M, Schwartz M, Larsen PL, Rosenberg T (2012) Phenotypic and genetic spectrum of Danish patients with ABCA4-related retinopathy. Ophthalmic Genet 33: 225–231. 22229821 10.3109/13816810.2011.643441

[13] Neveling K, Collin RW, Gilissen C, van Huet RA, Visser L, et al (2012) Next generation genetic testing for retinitis pigmentosa. Human mutation 33: 963–972. 22334370 10.1002/humu.22045 PMC3490376

[14] Audo I, Bujakowska KM, Leveillard T, Mohand-Said S, Lancelot ME, et al (2012) Development and application of a next-generation-sequencing (NGS) approach to detect known and novel gene defects underlying retinal diseases. Orphanet journal of rare diseases 7: 8–24. 22277662 10.1186/1750-1172-7-8 PMC3352121

[15] Glockle N, Kohl S, Mohr J, Scheurenbrand T, Sprecher A, et al (2014) Panel-based next generation sequencing as a reliable and efficient technique to detect mutations in unselected patients with retinal dystrophies. European journal of human genetics. Eur J Hum Genet 22: 99–104. 23591405 10.1038/ejhg.2013.72 PMC3865404

[16] Ross MG, Russ C, Costello M, Hollinger A, Lennon NJ, et al (2013) Characterizing and measuring bias in sequence data. Genome Biol 14: R51. 23718773 10.1186/gb-2013-14-5-r51 PMC4053816

[17] Coffey AJ, Kokocinski F, Calafato MS, Scott CE, Palta P, et al (2011) The GENCODE exome: sequencing the complete human exome. Eur J Hum Genet 19: 827–831. 21364695 10.1038/ejhg.2011.28 PMC3137498

[18] Chacon-Camacho OF, Granillo-Alvarez M, Ayala-Ramirez R, Zenteno JC (2013) ABCA4 mutational spectrum in Mexican patients with Stargardt disease: Identification of 12 novel mutations and evidence of a founder effect for the common p.A1773V mutation. Exp Eye Res 109: 77–82. 23419329 10.1016/j.exer.2013.02.006

[19] Jaakson K, Zernant J, Kulm M, Hutchinson A, Tonisson N, et al (2003) Genotyping microarray (gene chip) for the ABCR (ABCA4) gene. Human mutation 22: 395–403. 14517951 10.1002/humu.10263

[20] Scholl HP, Kremers J, Vonthein R, White K, Weber BH (2001) L- and M cone-driven electroretinograms in Stargardt's macular dystrophy-fundus flavimaculatus. Invest Ophthalmol Vis Sci 42: 1380–1389. 11328755

[21] Baum L, Chan WM, Li WY, Lam DS, Wang PB, et al (2003) ABCA4 sequence variants in Chinese patients with age-related macular degeneration or Stargardt's disease. Ophthalmologica 217: 111–114. 12592048 10.1159/000068553

[22] Yang Z, Chen Y, Lillo C, Chien J, Yu Z, et al (2008) Mutant prominin 1 found in patients with macular degeneration disrupts photoreceptor disk morphogenesis in mice. J Clin Invest 118: 2908–2916. 18654668 10.1172/JCI35891 PMC2483685

[23] Kniazeva M, Chiang MF, Morgan B, Anduze AL, Zack DJ, et al (1999) A new locus for autosomal dominant stargardt-like disease maps to chromosome 4. Am J Hum Genet 64: 1394–1399. 10205271 10.1086/302377 PMC1377876

[24] Maw MA, Corbeil D, Koch J, Hellwig A, Wilson-Wheeler JC, et al (2000) A frameshift mutation in prominin (mouse)-like 1 causes human retinal degeneration. Hum Mol Genet 9: 27–34. 10587575 10.1093/hmg/9.1.27

[25] Permanyer J, Navarro R, Friedman J, Pomares E, Castro-Navarro J, et al (2010) Autosomal recessive retinitis pigmentosa with early macular affectation caused by premature truncation in PROM1. Invest Ophthalmol Vis Sci 51: 2656 2663. 20042663 10.1167/iovs.09-4857 PMC2868491

[26] Littink KW, Koenekoop RK, van den Born LI, Collin RW, Moruz L, et al (2010) Homozygosity mapping in patients with cone-rod dystrophy: novel mutations and clinical characterizations. Invest Ophthalmol Vis Sci 51: 5943–5951. 20554613 10.1167/iovs.10-5797 PMC3061516

[27] Zhang Q, Zulfiqar F, Xiao X, Riazuddin SA, Ahmad Z, et al (2007) Severe retinitis pigmentosa mapped to 4p15 and associated with a novel mutation in the PROM1 gene. Hum Genet 122: 293–299. 17605048 10.1007/s00439-007-0395-2

[28] Pras E, Abu A, Rotenstreich Y, Avni I, Reish O, et al (2009) Cone-rod dystrophy and a frameshift mutation in the PROM1 gene. Mol Vis 15: 1709–1716. 19718270 PMC2732717

[29] Michaelides M, Johnson S, Poulson A, Bradshaw K, Bellmann C, et al (2003) An autosomal dominant bull's-eye macular dystrophy (MCDR2) that maps to the short arm of chromosome 4. Invest Ophthalmol Vis Sci 44: 1657–1662. 12657606 10.1167/iovs.02-0941

